# Elephantiasis Nostra Verrucosa Complicated by Cellulitis and Hemorrhagic Bullae: A Rare and Severe Clinical Presentation

**DOI:** 10.7759/cureus.102148

**Published:** 2026-01-23

**Authors:** Amna Nadeem, Iqra Choudhry

**Affiliations:** 1 Internal Medicine, St. George's University School of Medicine, Saint George's, GRD; 2 Internal Medicine, Humboldt Park Health, Chicago, USA; 3 Podiatry, Humboldt Park Health, Chicago, USA

**Keywords:** case report, cellulitis, chronic lymphedema, elephantiasis nostras verrucosa, hemorrhagic bullae

## Abstract

Elephantiasis nostras verrucosa (ENV) is a rare and debilitating manifestation of chronic nonfilarial lymphedema characterized by progressive dermal fibrosis, hyperkeratosis, and papillomatous skin changes. It arises from long-standing lymphatic obstruction and is most commonly associated with risk factors such as obesity, venous insufficiency, recurrent soft tissue infections, and conditions impairing lymphatic drainage. The true prevalence of ENV is poorly defined due to its rarity, though limited institutional data suggest it is uncommon even among high-risk populations. While ENV typically presents as slowly progressive limb enlargement with characteristic skin changes, atypical features may indicate advanced disease or superimposed infection. We report the case of a 48-year-old male with chronic lower-extremity lymphedema complicated by recurrent cellulitis who developed progressive cutaneous changes consistent with evolving ENV and was further complicated by the appearance of hemorrhagic bullae. This uncommon finding raised concern for severe infection and potential vascular compromise. Management focused on treating acute bacterial cellulitis while addressing the underlying lymphatic dysfunction through aggressive conservative measures. This case highlights the importance of early recognition of atypical skin findings in chronic lymphedema and reinforces the central role of lymphedema control in preventing disease progression and serious complications.

## Introduction

Elephantiasis nostras verrucosa (ENV) is a severe, end-stage manifestation of chronic nonfilarial lymphedema caused by long-standing lymphatic obstruction and inflammation [1]. It is an uncommon condition, with no well-defined incidence or prevalence; most epidemiologic data are derived from isolated case reports and small case series, primarily from tertiary care centers [1,2]. ENV predominantly affects adults with long-standing secondary lymphedema and is most frequently reported in individuals with morbid obesity (mean BMI 55.8 in one institutional series) or chronic venous disease (present in 71% of patients in the same cohort) [2]. The condition is predominantly bilateral (86%) and typically involves the lower extremities, particularly the calves (81%) [2]. Clinically, ENV is characterized by progressive dermal fibrosis, hyperkeratosis, papillomatosis, and irreversible enlargement of the affected limb.

The condition most commonly arises in patients with long-standing lymphedema, most often related to obesity, the most frequently reported underlying risk factor, as well as chronic venous insufficiency, recurrent cellulitis or erysipelas, malignancy, or prior surgical or radiation-related disruption of lymphatic drainage [2,3]. Obesity contributes through increased lymphatic load, impaired lymphatic function, and chronic low-grade inflammation, all of which drive disease progression [4,5]. This obesity-related lymphatic dysfunction creates a vicious cycle in which impaired lymphatic clearance promotes subcutaneous fat deposition and fibrosis, further worsening lymphatic impairment.

Chronic venous insufficiency, identified in approximately 71% of patients in one institutional series, may represent an underrecognized contributor to ENV development [2]. Recurrent episodes of cellulitis or erysipelas, reported in up to 86% of cases, cause progressive lymphatic damage, reinforcing a self-perpetuating cycle of infection, lymphatic injury, and worsening lymphedema [2,6].

Although ENV typically progresses slowly over many years, new or rapidly evolving skin findings can signal superimposed infection or worsening local disease [1,2]. Hemorrhagic bullae, while not specific for necrotizing soft tissue infection, are uncommon in uncomplicated cellulitis and may reflect severe inflammation, vascular fragility, or advanced lymphatic and venous compromise [7,8]. Bulla formation can occur in cellulitis, particularly in the setting of lymphedema, but the presence of hemorrhagic bullae should raise concern for more severe infection, as they are significantly associated with necrotizing fasciitis and increased mortality [8]. In patients with ENV, the appearance of such lesions warrants careful clinical assessment, as they may indicate necrotizing infection requiring urgent surgical evaluation rather than simple cellulitis alone. We report a rare case of ENV complicated by recurrent cellulitis and hemorrhagic bullae, selected for publication because it illustrates an unusual cutaneous manifestation of advanced disease and emphasizes the importance of timely recognition, appropriate interpretation of skin findings, and comprehensive management of chronic lymphedema.

## Case presentation

**Figure 1:**
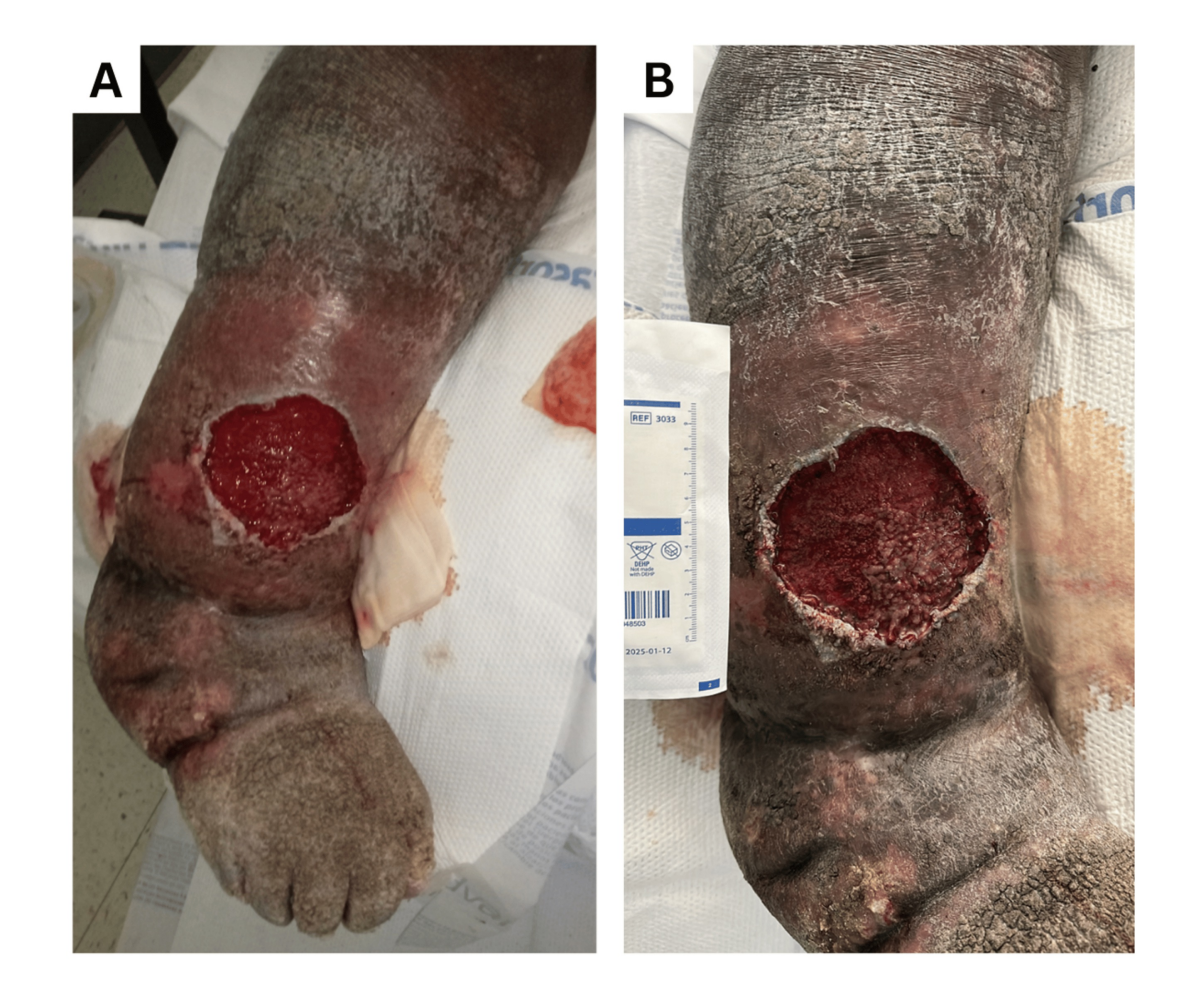
Clinical photographs of the left lower extremity. (A) The leg on admission with chronic lymphedema, verrucous skin changes, and an ulcer resulting from ruptured hemorrhagic bulla. (B) The leg after initial debridement of the ulcer.

A 48-year-old male with chronic bilateral lower-extremity lymphedema (BMI 38.6) presented with worsening swelling, erythema, and pain of the right lower extremity, which was more severely affected than the left. He had experienced three prior episodes of right-leg cellulitis over the past two years, treated intermittently with oral cephalexin or clindamycin. On examination, the right leg demonstrated marked nonpitting edema, hyperkeratosis, early verrucous changes, and a single tense hemorrhagic bulla measuring 8.5 cm in diameter and 6 mm deep (Figure 1). The lesion was erythematous, warm, and tender. Distal pulses were palpable, and the patient was hemodynamically stable.

Laboratory evaluation revealed leukocytosis (14,500/µL), anemia (Hgb 7 g/dL, Hct 21.8%), elevated CRP (27 mg/L), hypoalbuminemia, and elevated creatinine; coagulation studies were normal. Blood cultures were negative. Wound culture from the bulla grew Proteus mirabilis resistant to ciprofloxacin and TMP-SMX, as well as MRSA, and surgical pathology from soft tissue debridement showed skin and soft tissue gangrene with severe acute inflammation and abscess formation. X-rays of the tibia, fibula, and ankle showed no acute osseous abnormality, helping to exclude osteomyelitis.

The patient was treated with IV vancomycin and piperacillin-tazobactam for six days, followed by oral linezolid for one week. The hemorrhagic bulla was debrided, and tissue was submitted for culture and pathology. Multidisciplinary care included physical therapy for gait and strength, podiatric wound care with pulse lavage, topical betamethasone, clotrimazole, calmoseptine, vitamins A and D ointment, and multilayer compression dressings. Infectious disease and general surgery were consulted; surgery was deferred after ruling out necrotizing fasciitis and compartment syndrome. The patient was discharged with close outpatient follow-up and ongoing management of chronic lymphedema, including compression therapy, limb elevation, and meticulous skin care.

## Discussion

This case describes an advanced presentation of ENV in a patient with chronic lymphedema, obesity, and recurrent cellulitis, complicated by the rapid rupture and ulceration of a large hemorrhagic bulla. ENV represents an end-stage consequence of prolonged nonfilarial lymphatic obstruction and is most often associated with obesity, chronic venous insufficiency, and repeated soft-tissue infections [9]. Common complications include recurrent cellulitis or erysipelas, chronic ulceration, progressive verrucous skin changes, and, in severe cases, osteomyelitis or limb-threatening disease [9,10].

Hemorrhagic bullae are not specific markers of severe or necrotizing infection and may occur in uncomplicated cellulitis as a result of intense inflammation, vascular fragility, and impaired lymphatic or venous drainage [11]. However, in the setting of advanced ENV, large or rapidly evolving bullous lesions warrant careful evaluation to exclude deeper soft-tissue involvement and to assess disease severity. In this patient, laboratory studies demonstrated leukocytosis and elevated inflammatory markers without systemic instability, blood cultures were negative, and imaging showed no osseous involvement. These findings, along with surgical consultation, helped exclude necrotizing soft-tissue infection and supported a conservative management approach.

Microbiologic cultures identified *Proteus mirabilis* and MRSA, allowing for targeted antimicrobial therapy. The patient was treated with broad-spectrum intravenous antibiotics followed by oral therapy, consistent with management of severe cellulitis in the context of chronic lymphedema. Despite multiple prior antibiotic courses, infections recurred, reinforcing a key principle in ENV management: antimicrobial therapy alone is insufficient without addressing underlying lymphatic dysfunction. Conservative measures-including compression therapy, meticulous skin care, limb elevation, and weight reduction-remain the cornerstone of treatment and are supported by existing literature as effective strategies to reduce recurrence and slow disease progression [9,12].

Surgical intervention is generally reserved for refractory or function-limiting disease and is not first-line therapy [13]. In this case, interdisciplinary discussion favored continued medical and wound-based management after exclusion of surgical emergencies. This report adds to the limited literature by documenting an uncommon bullous complication in advanced ENV and underscores the importance of objective evaluation, appropriate interpretation of skin findings, and comprehensive lymphedema management to reduce morbidity.

## Conclusions

ENV is an uncommon end-stage complication of chronic nonfilarial lymphedema, with relatively few cases described in the literature. While cellulitis is a well-recognized complication of ENV, detailed descriptions of complex skin changes such as bullae followed by ulceration remain limited. Bullous lesions and ulcers can occur in uncomplicated cellulitis; however, their development in the setting of advanced ENV adds diagnostic and management challenges and warrants careful clinical evaluation. The primary value of this case lies in its contribution to the small but growing body of literature on ENV itself. Because ENV is rare, individual case reports remain important for improving understanding of its clinical course, associated complications, and management strategies. This case highlights how bullous and ulcerative changes may evolve in patients with longstanding lymphatic dysfunction and underscores the importance of objective assessment to differentiate between inflammatory, infectious, and ischemic processes.

Consistent with existing evidence, effective management requires a multidisciplinary approach that prioritizes long-term control of lymphatic stasis through compression therapy, meticulous skin care, and risk-factor modification, alongside appropriately targeted antimicrobial therapy when infection is present. Continued reporting of similar cases is essential to expand the limited clinical data available and to better inform the diagnosis and management of this challenging condition.
